# Factors associated with reactogenicity to an investigational HIV vaccine regimen in HIV vaccine trials network 702

**DOI:** 10.1016/j.vaccine.2024.05.039

**Published:** 2024-08-13

**Authors:** Rachel Chihana, Jia Jin Kee, Zoe Moodie, Yunda Huang, Holly Janes, Sufia Dadabhai, Alison C. Roxby, Mary Allen, Sheetal Kassim, Vimla Naicker, Craig Innes, Nivashnee Naicker, Thozama Dubula, Nicole Grunenberg, Mookho Malahleha, James G. Kublin, Linda-Gail Bekker, Glenda Gray, Johnstone Kumwenda, Fatima Laher

**Affiliations:** a Johns Hopkins Research Project, P.O Box 1131, Blantyre, Malawi; b Fred Hutchinson Cancer Center, P.O Box 19024, Seattle, WA 98109-1024, USA; c University of Washington, P.O Box 355852, WA 98195-5852, USA; d Fred Hutchinson Cancer Center, P.O Box 19024, WA 98109-1024, Seattle, USA; e National Institutes of Health, 9000, Rockville Pike, Bethesda, USA; f The Desmond Tutu HIV Centre, P.O Box 13801, Mowbray 7705, Cape Town, South Africa; g South Africa Medical Research Council, P.O Box 19070, Cape Town, South Africa; h Aurum Institute, The Ridge, 29 Queens Road, Johannesburg, South Africa; i Centre for the AIDS Programme of Research in South Africa (CAPRISA), University of KwaZulu–Natal, Private Bag X7, Durban, South Africa; j Walter Sisulu University, P. O Box 142, Eastern Cape, South Africa; k Setshaba Research Centre, P.O Box 468, Pretoria, South Africa; l Synergy Biomed Research Institute, 280 Oxford Street, East London 5201, South Africa; m Perinatal HIV Research Unit, P.O Box 114, University of the Witwatersrand, South Africa

**Keywords:** Reactogenicity, Vaccine, HIV, Age, Gender, Region

## Abstract

**Background:**

Reactogenicity informs vaccine safety, and may influence vaccine uptake. We evaluated factors associated with reactogenicity in HVTN 702, a typical HIV vaccine efficacy trial with multiple doses and products.

**Methods:**

HVTN 702, a phase 2b/3 double-blind placebo-controlled trial, randomized 5404 African participants aged 18–35 years without HIV to placebo, or ALVAC-HIV (vCP2438) at months 0, 1 and ALVAC-HIV (vCP2438) + Bivalent Subtype C gp120/MF59 at months 3, 6, 12 and 18. Using multivariate logistic regression, we evaluated associations between reactogenicity with clinical, sociodemographic and laboratory variables.

**Results:**

More vaccine than placebo-recipients reported local symptoms (all p < 0.001), arthralgia (p = 0.008), chills (p = 0.012) and myalgia (p < 0.001). Reactogenicity was associated with female sex at birth (OR_v_ = 2.50, OR_p_ = 1.81, both p < 0.001) and geographic region. Amongst vaccine-recipients, each year of age was associated with 3 % increase in reactogenicity (OR = 1.03, p = 0.002).

**Conclusion:**

Vaccine receipt, female sex at birth, older age, and region may affect reactogenicity.

## Highlights

• Those assigned female at birth experience more reactogenicity.
• Most symptoms are mild.
• Pain and/or tenderness symptom common in both vaccine and placebo groups.

## 1 Introduction

Reactogenicity encompasses the short-term subjective and objective physical manifestations of the inflammatory response to vaccination. Local reactogenicity symptoms include reported pain, and objective measurements of redness, and swelling at the injection site. Systemic reactogenicity symptoms include fever, and subjective reports of fatigue, headache, myalgia, arthralgia, chills and nausea [1].

Reactogenicity contributes to negative attitudes and reduced uptake of vaccines [2]. In one systematic review, safety and side effects concerns (including the life-threatening and severe, like thrombotic thrombocytopenia syndrome, myocarditis and pericarditis) affected COVID-19 vaccine acceptance [3]. Multiple factors influence reactogenicity. A study of health-sector employees found that female sex and Hispanic ethnicity were associated with higher odds of reporting systemic reactogenicity symptoms after a COVID-19 vaccine than male sex and other ethnicities [4]. Limited reactogenicity data exist for African adults due to few vaccination programs.

As the search for an efficacious HIV vaccine continues, elucidating factors associated with reactogenicity may help researchers optimize safety and tolerability. HVTN 702, demonstrated that although the vaccine regimen induced antibody and/or cellular immune responses in about two-thirds of vaccine-recipients, there was no protective efficacy [5].Very few HVTN 702 participants discontinued study product due to related adverse events [6]; therefore analysis of participant characteristics associated with these events was not conducted. Nonetheless, reactogenicity was reported by about one-third of placebo-recipients and 46 % of vaccine-recipients [6]. We investigated clinical, sociodemographic and laboratory variables associated with reactogenicity in HVTN 702.

## 2 Methods

### 2.1 Trial design

This was an ancillary analysis of data from HVTN 702 (NCT02968849), a multicenter, phase 2b/3 randomized, double-blind, placebo-controlled trial of a preventive HIV vaccine regimen which included i) ALVAC-HIV (vCP2438), a preparation of live, attenuated recombinant canarypox-derived virus expressing products from HIV-1: envelope ZM96 glycoprotein120 (clade C), envelope glycoprotein 41 TM (clade B), gag (clade B) and protease (clade B) and (ii) the Bivalent Subtype C glycoprotein 120/MF59 consisting of two subtype C recombinant monomeric proteins (TV1.C glycoprotein 120 envelope and 1086.C) and the MF59 adjuvant [6]. Participants were randomized 1:1 to ALVAC-HIV (vCP2438) at months 0 and 1 and ALVAC-HIV (vCP2438) plus Bivalent Subtype C glycoprotein 120/MF59 at months 3, 6, 12 and 18 (n = 2704); or to saline placebo (n = 2700) at all-time points [6].

### 2.2 Study participants

Between 2016 and 2019, HVTN 702 enrolled 5404 adults without HIV infection, aged 18–35 years from 14 sites in South Africa. This ancillary analysis included all participants who received at least one injection of vaccine or placebo. We analyzed by treatment received, not by randomization allocation, to account for rare errors in product administration.

### 2.3 Outcome variables

Post-vaccination, participants were observed for 30 min in clinic, trained to use a paper tool to log symptoms and instructed to present to clinic if concerning symptoms arose. After 3 days, researchers contacted participants to record reactogenicity data, and followed symptoms until resolution. Participants who self-reported a grade of greater than “mild” were seen by a clinician within 48 h.

Solicited local symptoms included pain and/or tenderness proximal to the injection site, erythema/redness and induration (grade 1: 2.5 − <5 cm [cm]/6.25 − <25 cm^2^ surface area (SA), grade 2: 5- <10 cm/25- < 100 cm^2^ SA, grade 3: ≥10 cm/≥100 cm^2^ SA or complications, grade 4: Potentially life-threatening complications). Solicited systemic symptoms included increased body temperature (≥38.0℃), malaise and/or fatigue, myalgia, headache, chills, arthralgia, nausea and vomiting.

The number and percentage of participants who experienced each reactogenicity sign or symptom were summarized by treatment received and grade (none/non gradable, mild/grade 1, moderate/grade 2, severe/grade 3 and life-threatening/grade 4). Each participant’s reactogenicity sign or symptom was counted once under the maximum severity for all injection visits.

These clinical, sociodemographic and laboratory test result characteristics were summarized by treatment received: age at randomization, age category at randomization (18–21, 22–25, 26–35 years), sex assigned at birth, gender identity (female, male, other), sexual orientation, race, body mass index (BMI), BMI category, number of live births (for participants with childbearing potential), geographic region, education level, married/have main partner status, urban living area, formal dwelling, and sexually transmitted infection (STI) status at baseline (available for participants enrolled after protocol version 2.0). Univariate and multivariate logistic regression were performed to assess associations between reactogenicity and sociodemographic and laboratory variables. Kruskal-Wallis tests were used to test for differences in reactogenicity severity between groups.

### 2.4 Ethical considerations

Research ethics committees of the Universities of the Witwatersrand, Cape Town, KwaZulu-Natal, Sefako Makgatho, and South African Medical Research Council approved the HVTN 702 trial. All study participants signed an informed consent document that provided study purpose, duration, procedures, alternatives, risks and benefits.

## 3 Results

### 3.1 Baseline characteristics

In HVTN 702, 2706 participants received the investigational vaccine, and 2698 received placebo. Vaccine and placebo groups had similarly distributed baseline characteristics (Appendix 1).

### 3.2 Reactogenicity

#### 3.2.1 Local reactogenicity

Of 5404 participants, 1020 (18.9 %) reported at least one local symptom. Pain and/or tenderness was the commonest in both groups; reported by 23.0 % (623/2706) of vaccine-recipients and 7.6 % (206/2698) of placebo-recipients. Most symptoms were mild (Appendix 1). Significantly more vaccine than placebo-recipients reported local reactogenicity symptoms across severity spectrum (p < 0.001 for all symptoms [Appendix 1]).

#### 3.2.2 Systemic reactogenicity

Of 5404 participants enrolled, 1642 (30.4 %) reported at least one systemic reactogenicity symptom. Headache was most frequent, experienced by 438/2706 (16.2 %) of vaccine-recipients and 423/2698 (15.7 %) of placebo-recipients (Appendix 1). Significant differences in the distribution of systemic reactogenicity between study groups were seen for arthralgia (p = 0.008), chills (p = 0.012) and myalgia (p < 0.001). Most subjective systemic symptoms were categorized as mild (Appendix 1) and none were grade 4. Fever was reported in 10.9 % of participants overall balanced between vaccine and placebo groups, with < 1.0 % (n = 6) experiencing grade 4 fever. Data were complete for all variables except fever, with measurement missing for 23 participants 0.4 %, with 14, 0.5 % for placebo and 14,03 % for vaccine-recipients.

#### 3.2.3 Reactogenicity by demographics

For both groups, those assigned female more frequently reported reactogenicity of any kind than those assigned male at birth regardless of treatment assignment. In the vaccine-recipients, over half of those assigned female 986/1895 (52.0 %) experienced reactogenicity compared to 267/811 (33.0 %) of those assigned male at birth. Similarly, in the placebo-recipients, more than one-third 682/1891 (36.0 %) of those assigned female experienced reactogenicity compared to 202/807 (25.0 %) of those assigned male at birth (Appendix 1). Participants with higher BMI (≥30 kg/meter^2^) experienced more frequent reactogenicity of any kind, with over half (363/659 [55.0 %]) of vaccine-recipients and over one-third (263/675 [39.0 %]) placebo participants reporting reactogenicity. More participants from Western/Eastern Cape province than other regions experienced reactogenicity; 301/503 (60.0 %) vaccine-recipients and 237/495 (48.0 %) placebo-recipients reported symptoms compared to 282/878 (32.0 %) vaccine-recipients and 178/886 (20.0 %) placebo-recipients from KwaZulu–Natal; and 670/1325 (51.0 %) vaccine-recipients and 469/1317 (36.0 %) placebo-recipients from the Central region. There were few grade 3 cases, especially in vaccine-recipients, and were mostly local rather than systemic reactogenicity (Appendix 1). There were no reports of grade 4 local reactogenicity.

In the multivariate analysis (Appendix 1), frequency of reactogenicity was 2.5 times higher in those assigned female than those assigned male at birth in vaccine-recipients (OR = 2.50, p < 0.001) and almost 2 times higher in placebo-recipients (OR 1.81, p < 0.001), after accounting for other variables (age, BMI, region, education, main sex partner, type of living area, baseline STI status, complete blood count [CBC] status). For vaccine-recipients only, after accounting for the same variables, every additional year of age was associated with a 3.0 % increase in frequency of reporting reactogenicity (OR = 1.03, p = 0.002). Region was associated with different reactogenicity in vaccine and placebo groups, after adjusting for the same variables, with 1.4 times more frequent reactogenicity reported by vaccine-recipients from Cape than vaccine-recipients from central provinces (OR 1.40, p = 0.008) and 1.3 times higher among placebo-recipients from Cape than central provinces (OR = 1.33, p = 0.028). Those in KwaZulu-Natal reported reactogenicity significantly less frequently than those in central provinces in both (vaccine OR = 0.46, p < 0.001, placebo OR = 0.42, p < 0.001) groups.

## 4 Discussion

In this ancillary analysis, those assigned female sex at birth, vaccine-recipients with increasing age and those living in the Cape provinces reported any reactogenicity more frequently. Overall, the vaccine was well-tolerated with mostly mild symptoms; the commonest were local pain and/or tenderness and headache. Both subjective and objective symptoms were more frequent in vaccine-recipients.

Findings are consistent to the RV144 trial with similar products, ALVAC-HIV and AIDSVAX B/E, in which pain and tenderness was the commonest local reactogenicity and symptoms were mostly mild to moderate [7] while mostly mild for HVTN 702. For both trials, moderate to severe local and systemic reactogenicity were more common in vaccine-recipients than placebo-recipients.

More participants assigned female, regardless of treatment assignment, reported reactogenicity than participants assigned male at birth. This is expected because compared to those assigned male, vaccine-recipients assigned female sex at birth experience more vaccine adverse events [1], [8], [9], [10], [11]. Females often develop higher vaccine-induced antibody titres than men, influenced by genetic and hormonal differences [9]. Sex hormones affect immune responses and cytokine levels, with oestrogens being immunostimulant and androgens being relatively immunosuppressive [10], [12], [13]. Females also develop greater immune responses to infection [14], [15].

Adults older than 60 years have reduced reactogenicity and immune responses to immunization than younger people [16]. However, in this group of participants aged 18–35 years, we saw small but statistically significant increases in reactogenicity with increasing age. Our study suggests that age-related reactogenicity patterns may differ by vaccine. However, more research is needed into whether increasing age correlates with increased reactogenicity for other types of HIV vaccines.

We found similar regional differences in reactogenicity in both groups, with more reactogenicity in the Western/Eastern Cape provinces than other regions. Geographic location may indirectly affect reactogenicity by influencing immune responses through socio-economic dynamics, genetics, climate, sun exposure, nutrition, and/or comorbidities [17]. There may have been differences in administration of the vaccine, although unlikely given standardized study staff training and monitoring. There may be social, behavioral and cultural differences in illness experiences post-vaccination [1].

Future HIV vaccine trials are likely to continue to examine sequential, multi-dose regimens with differing formulations over time. The experience in this trial suggests that local and systemic reactogenicity symptoms to similar regimens are to be expected in approximately 20 % of the adult population. Placebo-recipients report a substantial number of reactogenicity symptoms; trial participants should be counseled that they cannot predict their treatment assignment from reactogenicity [7], [11].

We analyzed one vaccine regimen and thus cannot yet generalize our findings about the associations between age, and sex at birth, and reactogenicity. However, our findings are supported by research on non-HIV vaccines: some – though not all – studies observed that women reported more reactogenicity than men from human papillomavirus (HPV) and H1N1 influenza vaccines [1] Reports about regional variation in HIV vaccine reactogenicity are scarce, and it is still unclear if, as we suspect, region is a proxy indicator for other phenomena. Ongoing research is assessing whether the same patterns of reactogenicity can be extrapolated to other HIV vaccine regimens.

We did not analyze findings by subjective versus objective reactogenicity, nor by the presence of adjuvants in the vector vaccine or not, nor by total number of injections over time, given the six-injection regimen.

Placebo and multi-dose, extended vaccine injections in the HVTN 702 trial for HIV prevention were well tolerated and most local and systemic reactogenicity was mild. Common symptoms like headache were reported similarly regardless of the treatment received. All participants in vaccine trials of similar agents should continue to be informed about possible reactogenicity.

## 5 Author confirmation

All authors attest they meet the ICMJE criteria for authorship.

## Funding

Funding was provided to Novartis Vaccines and Diagnostics (now part of the GlaxoSmithKline SA) by 10.13039/100000060NIAID (HHSN272201300033C//HHSN272201600012C) for the selection and process development of the two gp120 envelope proteins TV1.C and 1086.C, and by the 10.13039/100000865Bill & Melinda Gates Foundation Global Health Grant OPP1017604 and NIAID for the manufacture and release of the gp120 clinical grade material. GlaxoSmithKline Biologicals SA has contributed financially to provision of pre-exposure prophylaxis for participants. GlaxoSmithKline Biologicals SA was provided the opportunity to review a preliminary version of this manuscript for factual accuracy, but authors are solely responsible for final content and interpretation.

## CRediT authorship contribution statement

**Rachel Chihana:** Writing – original draft, Investigation, Formal analysis, Data curation, Conceptualization. **Jia Jin Kee:** Writing – review & editing, Methodology, Formal analysis, Data curation. **Zoe Moodie:** Writing – review & editing, Supervision, Methodology, Investigation, Formal analysis, Data curation. **Yunda Huang:** Writing – review & editing, Formal analysis. **Holly Janes:** Writing – review & editing, Formal analysis. **Sufia Dadabhai:** Writing – review & editing, Supervision, Methodology, Investigation, Conceptualization. **Alison C. Roxby:** Writing – review & editing, Supervision, Project administration, Methodology, Investigation, Formal analysis, Data curation, Conceptualization. **Mary Allen:** Writing – review & editing, Supervision. **Sheetal Kassim:** Writing – review & editing. **Vimla Naicker:** Writing – review & editing. **Craig Innes:** Writing – review & editing. **Nivashnee Naicker:** Writing – review & editing. **Thozama Dubula:** Writing – review & editing. **Nicole Grunenberg:** Writing – review & editing. **Mookho Malahleha:** Writing – review & editing. **James G. Kublin:** Writing – review & editing. **Linda-Gail Bekker:** Writing – review & editing. **Glenda Gray:** Writing – review & editing. **Johnstone Kumwenda:** Writing – review & editing, Supervision, Methodology, Investigation, Conceptualization. **Fatima Laher:** Writing – review & editing, Supervision, Formal analysis.

## Declaration of competing interest

The authors declare that they have no known competing financial interests or personal relationships that could have appeared to influence the work reported in this paper.

## Figures and Tables

**Table 1:** Baseline characteristics of study participants in HVTN 702 according to treatment received.

**Characteristic**	**N (%) or Median (min, max)**		
	**Total (n = 5404)**	**Vaccine (n = 2706)**	**Placebo (n = 2698)**
**Age in years at randomization**			
	24 (18, 35)	24 (18, 35)	24 (18, 35)
**Age category (in years) at randomization in years**			
18–21	1436 (27.0)	709 (26.0)	727 (27.0)
22–25	1858 (34.0)	944 (35.0)	914 (34.0)
26–35	2110 (39.0)	1053 (39.0)	1057 (39.0)
**Sex assigned at birth**			
Female	3786 (70.0)	1895 (70.0)	1891 (70.0)
**Gender identity**			
Female	3789 (70.0)	1899 (70.0)	1890 (70.0)
Male	1600 (30.0)	802 (30.0)	798 (30.0)
Another gender identity	15 (0.3)	5 (0.2)	10 (0.4)
**Sexual orientation**			
Heterosexual	5119 (95.0)	2552 (94.0)	2567 (95.0)
Another orientation	285 (5.0)	154 (6.0)	131 (5.0)
**Race**			
Asian	1 (0.0)	0 (0.0)	1 (0.0)
Black	5348 (99.0)	2674 (99.0)	2674 (99.0)
Colored/Mixed	40 (1.0)	23 (1.0)	17 (1.0)
Indian	2 (0.0)	1 (0.0)	1 (0.0)
White	6 (0.1)	3 (0.1)	3 (0.1)
Multiple	7 (0.1)	5 (0.2)	2 (0.1)
**Body Mass Index**			
Median (Min, Max)	24 (14, 59)	24 (15, 59)	24 (14, 59)
Body Mass Index category			
< 18.5 kg/m^2^	365 (7.0)	170 (6.0)	195 (7.0)
18.5 − < 25 kg/m^2^	2533 (47.0)	1285 (47.0)	1248 (46.0)
25 − < 30 kg/m^2^	1172 (22.0)	592 (22.0)	580 (21.0)
> =30 kg/m^2^	1334 (25.0)	659 (24.0)	675 (25.0)
**Region at enrollment**			
Central	2642 (49.0)	1325 (49.0)	1317 (49.0)
KwaZulu-Natal	1764 (33.0)	878 (32.0)	886 (33.0)
Western/Eastern Cape	998 (18.0)	503 (19.0)	495 (18.0)
**Education status**			
Primary or less	89 (2.0)	43 (2.0)	46 (2.0)
Secondary	4840 (90.0)	2411 (89.0)	2429 (90.0)
Post-secondary	473 (9.0)	252 (9.0)	221 (8.0)
Prefer not to answer	2 (0.04)	0 (0.0)	2 (0.07)
Married or has main sex partner*			
Yes	4725 (87.0)	2370 (88.0)	2355 (87.0)
No	421 (8.0)	220 (8.0)	201 (7.0)
Missing	258 (5.0)	116 (4.0)	142 (5.0)
Urban living area*			
Yes	4378 (81.0)	2203 (81.0)	2175 (81.0)
No	776 (14.0)	391 (14.0)	385 (14.0)
Missing	250 (5.0)	112 (4.0)	138 (5.0)
Formal dwelling*			
Yes	4192 (78.0)	2122 (78.0)	2070 (77.0)
No	962 (18.0)	472 (17.0)	490 (18.0)
Missing	250 (5.0)	112 (4.0)	138 (5.0)
Any STI at baseline**			
Positive	1241 (23.0)	637 (24.0)	604 (22.0)
Negative	3397 (63.0)	1698 (63.0)	1699 (63.0)
Not done/indeterminate	3 (0.1)	1 (0.0)	2 (0.1)
Missing	763 (14.0)	370 (14.0)	393 (15.0)
Number of babies alive at birth among participants assigned female at birth*	**N = 3786**	**N = 1895**	**N = 1891**
0	900 (24.0)	452 (24.0)	448 (24.0)
1	1748 (46.0)	882 (47.0)	866 (46.0)
> =2	945 (25.0)	474 (25.0)	471 (25.0)
Missing	193 (5.0)	87 (5.0)	106 (6.0)
**Abnormal CBC at baseline**	**N = 5404**	**N = 2706**	**N = 2698**
Yes	116 (2.0)	52 (2.0)	64 (2.0)
No	5256 (97.0)	2640 (98.0)	2616 (97.0)
Missing	32 (1.0)	14 (1.0)	18 (1.0)

* Question introduced after study began and asked retrospectively when required. It is missing for participants who were lost to follow up prior to its introduction.

** Sexually transmitted infections (Syphilis, Chlamydia/Gonorrhea, Trichomonas Vaginalis) at baseline only available on participants enrolled under protocol version 2.0 and higher.

**Table 2:** Comparison of local and systemic reactogenicity symptoms by treatment received in the HVTN 702 trial.

**Characteristic**	**N (%)**			
	**Total (N = 5404)**	**Vaccine (N = 2706)**	**Placebo (N = 2698)**	*P − value
***Local symptoms***				
**Erythema/Redness**				< 0.001
None / Not Gradable	5177 (95.8)	2496 (92.2)	2681 (99.4)	
Grade 1	122 (2.3)	108 (4.0)	14 (0.5)	
Grade 2	77 (1.4)	74 (2.7)	3 (0.1)	
Grade 3	28 (0.5)	28 (1.0)	0 (0.0)	
Grade 4	0 (0.0)	0 (0.0)	0 (0.0)	
**Induration/Swelling**				< 0.001
None / Not Gradable	5055 (93.5)	2382 (88.0)	2673 (99.1)	
Grade 1	175 (3.2)	159 (5.9)	16 (0.6)	
Grade 2	140 (2.6)	133 (4.9)	7 (0.3)	
Grade 3	34 (0.6)	32 (1.2)	2 (0.1)	
Grade 4	0 (0.0)	0 (0.0)	0 (0.0)	
**Erythema and/or Induration**				< 0.001
None / Not Gradable	4976 (92.1)	2316 (85.6)	2660 (98.6)	
Grade 1	222 (4.1)	196 (7.2)	26 (1.0)	
Grade 2	166 (3.1)	156 (5.8)	10 (0.4)	
Grade 3	40 (0.7)	38 (1.4)	2 (0.1)	
Grade 4	0 (0.0)	0 (0.0)	0 (0.0)	
**Pain**				< 0.001
None	4700 (87.0)	2163 (79.9)	2537 (94.0)	
Grade 1	629 (11.6)	480 (17.7)	149 (5.5)	
Grade 2	74 (1.4)	62 (2.3)	12 (0.4)	
Grade 3	1 (0.0)	1 (0.0)	0 (0.0)	
Grade 4	0 (0.0)	0 (0.0)	0 (0.0)	
**Tenderness**				< 0.001
None/ Not Gradable	4968 (91.9)	2369 (87.5)	2599 (96.3)	
Grade 1	388 (7.2)	298 (11.0)	90 (3.3)	
Grade 2	47 (0.9)	38 (1.4)	9 (0.3)	
Grade 3	1 (0.0)	1 (0.0)	0 (0.0)	
Grade 4	0 (0.0)	0 (0.0)	0 (0.0)	
**Pain and/or Tenderness**				< 0.001
None/ Not Gradable	4575 (84.7)	2083 (77.0)	2492 (92.4)	
Grade 1	746 (13.8)	553 (20.4)	193 (7.2)	
Grade 2	82 (1.5)	69 (2.5)	13 (0.5)	
Grade 3	1 (0.0)	1 (0.0)	0 (0.0)	
Grade 4	0 (0.0)	0 (0.0)	0 (0.0)	
***Systemic symptoms***				
**Arthralgia**				0.008
None/ Not Gradable	5271 (97.5)	2624 (97.0)	2647 (98.1)	
Grade 1	115 (2.1)	75 (2.8)	40 (1.5)	
Grade 2	18 (0.3)	7 (0.3)	11(0.4)	
Grade 3	0 (0.0)	0 (0.0)	0(0.0)	
Grade 4	0 (0.0)	0 (0.0)	0(0.0)	
**Chills**				0.012
None/ Not Gradable	5255 (97.2)	2616 (96.7)	2639(97.8)	
Grade 1	138 (2.6)	87 (3.2)	51(1.9)	
Grade 2	11 (0.2)	3 (0.1)	8(0.3)	
Grade 3	0 (0.0)	0 (0.0)	0(0.0)	
Grade 4	0 (0.0)	0 (0.0)	0(0.0)	
**Headache**				0.597
None/ Not Gradable	4543 (84.1)	2268 (83.8)	2275(84.3)	
Grade 1	628 (11.6)	318 (11.8)	310(11.5)	
Grade 2	229 (4.2)	117 (4.3)	112(4.2)	
Grade 3	4 (0.1)	3 (0.1)	1(0.0)	
Grade 4	0 (0.0)	0 (0.0)	0(0.0)	
**Malaise and/or fatigue**				0.057
None/ Not Gradable	4958 (91.7)	2463 (91.0)	2495 (92.5)	
Grade 1	398 (7.4)	221 (8.2)	177 (6.6)	
Grade 2	45 (0.8)	21 (0.8)	24 (0.9)	
Grade 3	3 (0.1)	1 (0.0)	2 (0.1)	
Grade 4	0 (0.0)	0 (0.0)	0 (0.0)	
**Myalgia**				< 0.001
None/ Not Gradable	5248 (97.1)	2606 (96.3)	2642 (97.9)	
Grade 1	133 (2.5)	85 (3.1)	48 (1.8)	
Grade 2	23 (0.4)	15 (0.6)	8 (0.3)	
Grade 3	0 (0.0)	0 (0.0)	0 (0.0)	
Grade 4	0 (0.0)	0 (0.0)	0 (0.0)	
**Nausea**				0.905
None/ Not Gradable	5168 (95.6)	2587 (95.6)	2581 (95.7)	
Grade 1	211 (3.9)	104 (3.8)	107 (4.0)	
Grade 2	25 (0.5)	15 (0.6)	10 (0.4)	
Grade 3	0 (0.0)	0 (0.0)	0 (0.0)	
Grade 4	0 (0.0)	0 (0.0)	0 (0.0)	
**Temperature**				0.901
None: < 38.0C	4815 (89.1)	2410 (89.1)	2405 (89.1)	
Grade 1: 38.0– < 38.6C	344 (6.4)	168 (6.2)	176 (6.5)	
Grade 2: 38.6– < 39.3C	182 (3.4)	98 (3.6)	84 (3.1)	
Grade 3: 39.3– < 40.0C	34 (0.6)	20 (0.7)	14 (0.5)	
Grade 4: > = 40.0C	6 (0.1)	1 (0.0)	5 (0.2)	
Missing	23 (0.4)	9 (0.3)	14 (0.5)	
**Vomiting**				0.513
None/ Not Gradable	5254 (97.2)	2627 (97.1)	2627 (97.4)	
Grade 1	128 (2.4)	64 (2.4)	64 (2.4)	
Grade 2	22 (0.4)	15 (0.6)	7 (0.3)	
Grade 3	0 (0.0)	0 (0.0)	0 (0.0)	
Grade 4	0 (0.0)	0 (0.0)	0 (0.0)	
**Maximum Systemic Symptoms (excluding Temperature)**				0.004
None/ Not Gradable	4099 (75.9)	2005 (74.1)	2094 (77.6)	
Grade 1	1004 (18.6)	546 (20.2)	458 (17.0)	
Grade 2	294 (5.4)	151 (5.6)	143 (5.3)	
Grade 3	7 (0.1)	4 (0.1)	3 (0.1)	
Grade 4	0 (0.0)	0 (0.0)	0 (0.0)	

* P- value determined by Kruskal Wallis test.

**Table 3:** Description of reactogenicity by sociodemographic and laboratory factors according to treatment received in the HVTN 702.

**Characteristic**	**N with reactogenicity (%)**	
	**Vaccine (n = 2706)**	**Placebo (n = 2698)**
**Age category(years) at randomization**		
18–21	307 / 709 (43.0)	213 / 727 (29.0)
22–25	441 / 944 (47.0)	316 / 914 (35.0)
26–35	505 / 1053 (48.0)	355 / 1057 (34.0)
**Sex assigned at birth**		
Female	986 / 1895 (52.0)	682 / 1891 (36.0)
Male	267 / 811 (33.0)	202 / 807 (25.0)
**Gender identity**		
Female	989 / 1899 (52.0)	681 / 1890 (36.0)
Male	262 / 802 (33.0)	199 / 798 (25.0)
Another gender identity	2 / 5 (40.0)	4 / 10 (40.0)
**Sexual orientation**		
Heterosexual	1187 / 2552 (47.0)	842 / 2567 (33.0)
Another orientation	66 / 154 (43.0)	42 / 131 (32.0)
**Race**		
Black	1240 / 2674 (46.0)	877 / 2674 (33.0)
Colored/Mixed	10 / 23 (43.0)	5 / 17 (29.0)
White	1 / 3 (33.0)	1 / 3 (33.0)
Multiple	2 / 5 (40.0)	1 / 2 (50.0)
BMI category*		
< 18.5 kg/m^2^	55 / 170 (32.0)	61 / 195 (31.0)
18.5 − < 25 kg/m^2^	545 / 1285 (42.0)	381 / 1248 (31.0)
25 − < 30 kg/m^2^	290 / 592 (49.0)	179 / 580 (31.0)
> =30 kg/m^2^	363 / 659 (55.0)	263 / 675 (39.0)
Number of infants alive at birth among participants assigned female at birth**		
0	250 / 452 (55.0)	156 / 448 (35.0)
1	456 / 882 (52.0)	332 / 866 (38.0)
> =2	255 / 474 (54.0)	164 / 471 (35.0)
Missing	25 / 87 (29.0)	30 / 106 (28.0)
**Region at enrollment**		
Central	670 / 1325 (51.0)	469 / 1317 (36.0)
KwaZulu-Natal	282 / 878 (32.0)	178 / 886 (20.0)
Western/Eastern Cape	301 / 503 (60.0)	237 / 495 (48.0)
**Education status**		
Primary or less	11 / 43 (26.0)	15 / 46 (33.0)
Secondary	1106 / 2411 (46.0)	797 / 2429 (33.0)
Post-secondary	136 / 252 (54.0)	72 / 221 (33.0)
Married or has main sex partner**		
Yes	1110 / 2370 (47.0)	776 / 2355 (33.0)
No	111 / 220 (50.0)	70 / 201 (35.0)
Missing	32 / 116 (28.0)	38 / 142 (27.0)
Urban living area**		
Yes	1071 / 2203 (49.0)	744 / 2175 (34.0)
No	151 / 391 (39.0)	102 / 385 (26.0)
Missing	31 / 112 (28.0)	38 / 138 (28.0)
Formal dwelling**		
Yes	1003 / 2122 (47.0)	671 / 2070 (32.0)
No	219 / 472 (46.0)	175 / 490 (36.0)
Missing	31 / 112 (28.0)	38 / 138 (28.0)
Any STI at baseline***		
Positive	291 / 637 (46.0)	190 / 604 (31.0)
Negative	773 / 1698 (46.0)	522 / 1699 (31.0)
Not done/indeterminate	1 / 1 (100.0)	2 / 2 (100.0)
Missing	188 / 370 (51.0)	170 / 393 (43.0)
**Abnormal CBC at baseline**		
Yes	27 / 52 (52.0)	22 / 64 (34.0)
No	1221 / 2640 (46.0)	855 / 2616 (33.0)
Missing	5 / 14 (36.0)	7 / 18 (39.0)

* BMI = Body Mass Index.

** Question introduced after study began and asked retrospectively when required. It is missing for participants who were lost to follow up prior to its introduction.

*** Sexually transmitted infections at baseline was only available on participants enrolled under protocol version 2.0 and higher.

**Table 4:** Multivariate logistic regression analysis of sociodemographic and laboratory factors associated with reactogenicity among study participants who received vaccine or placebo in the HVTN 702.

**Characteristic**	**Vaccine**		**Placebo**	
	**Odds Ratio (95 % CI)**	**Nominal P**	**Odds Ratio (95 % CI)**	**Nominal P**
Age at randomization	1.03 (1.01, 1.06)	0.002	1.00 (0.98, 1.03)	0.723
Sex assigned at birth (Ref*: Male)				
Female	2.50 (1.98, 3.15)	< 0.001	1.81 (1.40, 2.35)	< 0.001
BMI**	1.01 (0.99, 1.02)	0.261	1.01 (1.00, 1.03)	0.090
Region at enrollment (Ref*: Central)				
KwaZulu-Natal	0.46 (0.37, 0.56)	< 0.001	0.42 (0.33, 0.52)	< 0.001
Western/Eastern Cape	1.40 (1.09, 1.79)	0.008	1.33 (1.03, 1.70)	0.028
Education status (Ref*: Primary or less)				
Secondary	1.81 (0.88, 3.97)	0.120	1.07 (0.53, 2.30)	0.863
Post-secondary	2.11 (0.97, 4.85)	0.067	1.01 (0.47, 2.29)	0.989
Married or has main sex partner (Ref*: No)				
Yes	0.85 (0.62, 1.15)	0.293	0.86 (0.61, 1.21)	0.384
Urban living area (Ref*: No)				
Yes	1.21 (0.95, 1.56)	0.128	1.28 (0.97, 1.69)	0.081
Formal dwelling (Ref*: No)				
Yes	1.13 (0.89, 1.44)	0.307	0.83 (0.65, 1.06)	0.129
Any STI at baseline (Ref*: Negative)				
Positive	0.86 (0.71, 1.05)	0.141	0.89 (0.72, 1.11)	0.310
Abnormal CBC*** at baseline (Ref*: No)				
Yes	0.97 (0.51, 1.82)	0.915	0.78 (0.44, 1.43)	0.416

Statistical method used was multivariate logistic regression adjusted for the following variables: age, body mass index, region, education, main sex partner, type of living area, baseline sexually transmitted infection status and complete blood count status.

* Ref = Reference.

** BMI = Body Mass Index.

*** CBC = Complete Blood Count.

## Data Availability

The authors do not have permission to share data.
